# Efficacy of common laboratory disinfectants and heat on killing trypanosomatid parasites

**DOI:** 10.1186/1756-3305-1-35

**Published:** 2008-09-22

**Authors:** Xia Wang, Momodou Jobe, Kevin M Tyler, Dietmar Steverding

**Affiliations:** 1 BioMedical Research Centre, School of Medicine, Health Policy and Practice, University of East Anglia, Norwich, NR4 7TJ, UK

## Abstract

The disinfectants TriGene, bleach, ethanol and liquid hand soap, and water and temperature were tested for their ability to kill bloodstream forms of *Trypanosoma brucei*, epimastigotes of *Trypanosoma rangeli *and promastigotes of *Leishmania major*. A 5-min exposure to 0.2% TriGene, 0.1% liquid hand soap and 0.05% bleach (0.05% NaOCl) killed all three trypanosomatids. Ethanol and water destroyed the parasites within 5 min at concentrations of 15–17.5% and 80–90%, respectively. All three organisms were also killed when treated for 5 min at 50°C. The results indicate that the disinfectants, water and temperature treatment (i.e. autoclaving) are suitable laboratory hygiene measures against trypanosomatid parasites.

## Findings

Demonstration of the efficacy of disinfectants against animal and human pathogens has become a requisite part of the documentation associated with licensed handling. In order to obtain a licence for working with trypanosomatid parasites, authorities request verification that the disinfectants and autoclaving conditions indicated in many standard operating procedures to inactivate the pathogens are indeed capable of efficient killing of the organisms. As such data has not been readily available either from manufacturers or as publication, in recent years each laboratory has been required to carry out inactivation experiments independently before further work can be undertaken. The purpose of this report is to confirm that disinfectants commonly used in laboratories and heat treatment result in killing of trypanosomatid parasites.

We tested the commercial disinfectant TriGene (MediChem International Ltd., U.K.), and bleach (sodium hypochlorite (NaOCl) solution; Fisher Scientific, U.K.) and ethanol as general laboratory disinfectants for their ability to kill bloodstream forms of *Trypanosoma brucei *(clone 427-221a [1]), epimastigotes of *Trypanosoma rangeli *(Choachi strain [2]) and promastigotes of *Leishmania major *(Friedlin strain [3]). In addition, we also investigated the effect of dilution in water, liquid hand soap (RBS HDS 10; Medline Scientific LTD., U.K.) and heat treatment on the parasites. The parasites were incubated at a cell density of 1 × 10^6^/ml with various concentrations of the reagents in appropriate medium (*T. brucei*, Baltz medium plus 20% heat-inactivated foetal calf serum (iFCS) [4]; *T. rangeli*, Liver Infusion Tryptose medium plus 10% iFCS [2]; *L. major*, medium 199 plus 10% iFCS [5]) in a final volume of 1 ml at room temperature. The controls contained the corresponding amount of water (except for experiments testing the effect of dilution in water where the controls contained only medium). After 5 min incubation, live cells were counted using a Neubauer haemocytometer. The 50% lethal concentration (LC_50_), i.e. the reagent concentration necessary to kill 50% of the cells compared to the control, was determined by linear interpolation [6]. The 100% lethal concentration (LC_100_), i.e. the lowest concentration of a reagent at which all cells were killed, was determined microscopically. For heat treatment, parasites at a cell density of 1 × 10^6^/ml in 1 ml appropriate medium were incubated at different temperature using a digital heater block (Grant Instruments, U.K.). Samples incubated at room temperature served as controls. After 5 min incubation, live cells were counted using a Neubauer haemocytometer. The 50% lethal temperature (LT_50_), i.e. the temperature necessary to kill 50% of the cells compared to the control, was determined by linear interpolation [6]. The 100% lethal temperature (LT_100_), i.e. the lowest temperature at which all cells were killed, was determined microscopically.

For TriGene, bleach and liquid hand soap, the same LC_100 _value was observed for all three parasites (Table 1). Based on LC_50 _values, *T. brucei *appears to be approximately 4-fold more resistant towards TriGene while *T. rangeli *are about 2-fold more sensitive towards liquid hand soap, compared with the other two parasites, respectively. Regarding ethanol and dilution in water, *T. rangeli *seems to be somewhat more resistant to these reagents than *T. brucei *and *L. major *(Table 1). The LC_100 _values for ethanol, TriGene and bleach are 4, 10 and 20 times higher than the recommended working concentrations of these disinfectants which are 70%, 2% and 1%, respectively. This shows that trypanosomatids are very sensitive to commonly used laboratory disinfectants. In the case of TriGene it has been shown that bloodstream forms of *T. brucei *are killed at a concentration of 0.1% within 20 s [7]. The finding that liquid hand soap efficiently destroys all three parasites suggests that soap solutions can be used as first aid measure to clean skin areas accidentally contaminated with the pathogens. The dilution experiment with water indicates that trypanosomatids cannot cope very well with hypoosmotic stress even though these parasites are capable of some kind of osmoregulation [8].

**Table 1:** LC_50 _and LC_100 _values of disinfectants and water for bloodstream forms of *T. brucei*, epimastigotes of *T. rangeli *and promastigotes of *Leishmania major*.

	*T. brucei*		*T. rangeli*		*L. major*	
	
Reagent	LC_50_	LC_100_	LC_50_	LC_100_	LC_50_	LC_100_
Bleach *	0.019	0.05	0.016	0.05	0.021	0.05
Trigene	0.134	0.2	0.037	0.2	0.029	0.2
Ethanol	10.6	15	13.2	17.5	10.9	15
Soap^†^	0.068	0.1	0.035	0.1	0.063	0.1
Water	64	80	72	90	65	90

LC_50 _and LC_100 _values are presented in %. Each value represents the mean of three independent experiments. Standard deviations were less than 10%. LC_50_, 50% lethal concentration; LC_100_, 100% lethal concentration.

* As NaOCl. In aqueous solutions, NaOCl forms NaOH and HOCl (hypochlorous acid). HOCl is the active reagent what kills pathogens and is referred to as available chlorine. At a pH of ~7 and at room temperature, 80% of the chlorine is in the available form (HOCl) [10]. For example, 0.05% bleach equals 0.05% NaOCl which produces around 0.04% HOCl.

^†^Liquid hand soap.

All three trypanosomatid parasites are equally sensitive to heat treatment (Table 2). No difference was observed for the LT_100 _and the LT_50 _varied only by 3°C. The finding that the parasites are already killed at 50°C indicates that trypanosomatids are very temperature sensitive and thus would certainly not survive normal autoclaving condition of 121°C and 1.4 bar for 15 min. Actually, it has been shown that cultures of bloodstream forms of *T. brucei *post autoclaving contained only cell debris and immotile, rounded-up cells [9].

**Table 2:** Effect of temperature on bloodstream forms of *T. brucei*, epimastigotes of *T. rangeli *and promastigotes of *L. major*.

Species	LT_50_	LT_100_
*T. brucei*	45	50
*T. rangeli*	42	50
*L. major*	44	50

LT_50 _and LT_100 _are presented in °C. Each value represents the mean of three independent experiments. The standard deviations were less than 2%. LT_50_, 50% lethal temperature; LT_100_, 100% lethal temperature.

In this study we have shown that bloodstream forms of *T. brucei*, epimastigotes of *T. rangeli *and promastigotes of *L. major *are quite fragile organisms which can be easily killed with disinfectants commonly used in laboratories and by heat treatment. All three parasite species exhibited very similar sensitivities for the reagents tested and temperature. As these three parasites are representatives of the Salivaria group (*T. brucei brucei*, *T. brucei rhodesiense*, *T. brucei gambiense*, *T. congolense *and *T. vivax*), the Stercoraria group (sibling species *T. cruzi *and *T. rangeli*) and the *Leishmania *genus (*L. major*, *L. donovani*, *L. infantum*, *L. mexicana*, *L. braziliensis*, *L amazonensis *etc.), our findings likely indicate that all other pathogenic trypanosomatids display similar susceptibilities for these disinfectant and temperature treatments. In conclusion, common laboratory disinfectant (at the indicated concentrations) and temperature treatment can be used for effective inactivation of waste liquid and general laboratory ware that has been contaminated with trypanosomatid parasites.

## Competing interests

The authors declare that they have no competing interests.

## Authors' contributions

XW, MJ and DS carried out the experiments. DS and KMT conceived the study, supervised the execution, and prepared the final draft of the manuscript. All authors have read and approved the final manuscript.
